# Mass Spectrometry-Based Proteomics of Human Milk to Identify Differentially Expressed Proteins in Women with Breast Cancer versus Controls

**DOI:** 10.3390/proteomes10040036

**Published:** 2022-10-28

**Authors:** Roshanak Aslebagh, Danielle Whitham, Devika Channaveerappa, Panashe Mutsengi, Brian T. Pentecost, Kathleen F. Arcaro, Costel C. Darie

**Affiliations:** 1 Biochemistry and Proteomics Laboratories, Department of Chemistry & Biomolecular Science, Clarkson University, Potsdam, NY 13699-5810, USA; 2 Department of Veterinary & Animal Sciences, University of Massachusetts, Amherst, MA 01003-9298, USA

**Keywords:** breast cancer, milk, proteomics, mass spectrometry, protein dysregulation, biomarkers

## Abstract

It is thought that accurate risk assessment and early diagnosis of breast cancer (BC) can help reduce cancer-related mortality. Proteomics analysis of breast milk may provide biomarkers of risk and occult disease. Our group works on the analysis of human milk samples from women with BC and controls to investigate alterations in protein patterns of milk that could be related to BC. In the current study, we used mass spectrometry (MS)-based proteomics analysis of 12 milk samples from donors with BC and matched controls. Specifically, we used one-dimensional (1D)-polyacrylamide gel electrophoresis (PAGE) coupled with nanoliquid chromatography tandem MS (nanoLC-MS/MS), followed by bioinformatics analysis. We confirmed the dysregulation of several proteins identified previously in a different set of milk samples. We also identified additional dysregulations in milk proteins shown to play a role in cancer development, such as Lactadherin isoform A, O-linked N-acetylglucosamine (GlcNAc) transferase, galactosyltransferase, recoverin, perilipin-3 isoform 1, histone-lysine methyltransferase, or clathrin heavy chain. Our results expand our current understanding of using milk as a biological fluid for identification of BC-related dysregulated proteins. Overall, our results also indicate that milk has the potential to be used for BC biomarker discovery, early detection and risk assessment in young, reproductively active women.

## 1. Introduction

BC is one of the most common cancers worldwide and in the United States [1,2,3]. Accurate risk assessment and earlier detection would benefit all women especially young women for whom mammography is not effective due to their dense breast tissue [4], and reproductively active women who might be temporarily at a higher risk of pregnancy-related BC [5,6]. A biomarker is a protein, set of proteins or other molecules whose dysregulation is consistently associated with a disease or disorder. One of the most robust and common tools for the discovery of protein biomarkers is MS, which is a precise method applied in identification, quantitation, characterization and post translational modifications of proteins [7]. Early diagnosis and risk assessment of BC could be achieved non-invasively by the discovery of BC biomarkers in different types of bodily fluids, and much research has been published on this subject [8,9]. Still, there remains a need for more research in this field to provide a comprehensive biomarker signature for BC based on the protein biomarkers found in bodily fluids. Human milk, directly derived from the breast ducts, has been studied for BC investigations [4,5,8,10,11,12,13] and is accepted as a proper microenvironment for the purpose of BC biomarker discovery [1,2,3,4,5,6,10,13,14]

We previously investigated protein dysregulations in 10 human milk samples, (from 5 women with BC and 5 controls) using 1D-SDS-PAGE coupled with nanoLC-MS/MS and identified several dysregulated (upregulated or downregulated) proteins [5]. In a second study we focused on one of these comparison pairs, a *within* woman comparison. Specifically, both samples (BC and control) were donated by the same woman, one from the breast identified with BC 24 months *after* donation, and one from the contralateral. We performed 2D-SDS-PAGE coupled with nanoLC-MS/MS to achieve a more comprehensive investigation of dysregulated proteins in this pair of samples and identified several dysregulated proteins [15]. Most of the proteins identified in our previous work have been shown to be potentially involved in cancer development and some have been reported to be dysregulated in either cancer or cancer cell lines (reviewed in our previous studies [5,15]. In the present study, we used 1D-SDS-PAGE coupled with nanoLC-MS/MS to analyze a new set of paired milk samples (n = 6 pairs). In the study, 5 of the 6 comparison pairs include BC vs. control pairs, 4 of which are *across* women comparisons, meaning that the BC sample is milk combined from left and right breasts of a woman diagnosed with BC compared to milk combined from left and right breasts of another woman with no cancer diagnosis. In addition, one, comparison pair is a *within* woman comparison, meaning that the BC sample came from the right breast of a woman diagnosed with cancer in the right breast and the control sample came from her unaffected left breast. We also analyzed one comparison pair from the right and left breasts of a woman without BC, to investigate the protein differences between the milk from two breasts. We applied 1D-SDS-PAGE coupled with nanoLC-MS/MS on these 6 pairs of human milk samples and we were able to identify several protein dysregulations (upregulations or downregulations) some of which were identified in our previous studies as well. These dysregulated proteins might be considered as potential future biomarkers for BC early detection and risk assessment.

## 2. Materials and Methods

### 2.1. Human Subjects and Milk Samples

Analyses were performed on 12 human milk samples collected with IRB approval from the University of Massachusetts, Amherst. The procedure for sample collection has been described elsewhere [10,13]. Briefly, milk samples received at the laboratory between 2008 and 2015 were aliquoted and maintained at −20 °C. We attempted to match cases and controls for mother’s age at sample donation and age at first birth, the number of live births, and the length of time samples were maintained at −20 °C (Table 1). The participants who donated milk and were diagnosed with BC comprised two categories: 1) they were diagnosed with BC *before* milk donation, or 2) they were diagnosed with BC *after* milk donation. Table 1 provides the participant demographics that were used for assigning the comparison pairs. As shown in Table 1, analyses were conducted on milk donated by 10 women. For 8 women (4 with BC and 4 controls) samples prepared by combining samples from right and left breasts were analyzed. These samples provided 4 comparison pairs with the following sample codes: 1_BC vs. 2_Con, 3_BC vs. 4_Con, 5_BC vs. 6_Con and 7_BC vs. 8_Con). The 9th woman provided two milk samples, one from the right breast diagnosed with cancer, and a control sample from the left breast, in which there was no cancer, allowing a *within* woman comparison (9_R_BC vs. 9_L_Con). Lastly, the 10th woman, who did not have BC, donated milk from her right and left breasts, allowing a *within* woman comparison of protein patterns from two control breasts (10_R_Con vs. 10_L_Con). As seen in Table 1, Sample 3_BC was donated 6.2 years *after* the participant was diagnosed with BC. We compared this sample with a milk sample from a woman who was never diagnosed with BC, to observe whether alterations in protein pattern remain years *after* the BC was removed.

Comparison pairs (BC versus control) were assigned in an attempt to minimize differences in BC risk factors including mother’s age, her age at first birth, and number of births. It was not possible to match BC and control samples on baby’s age. Comparison pairs were analyzed at the same time to minimize potential errors resulting from possible deviations in the performance of the instruments. Except for samples from participants 9 and 10 (milk samples 9_R_BC, 9_L_Con, 10_R_Con, 10_L_Con), all samples are mixtures of milk from the right and left breasts. For participant 9 (a woman with BC in the right breast) and 10 (a woman without BC), milk was taken separately from the right and left breasts, and the comparison was between the milk from right and left breasts.

### 2.2. Reagents

All the chemicals used in this study were from Sigma-Aldrich (St. Louis, MO, USA).

### 2.3. MS-Based Proteomics Analysis

As described in our previous study [5], the following procedure was followed for MS-based proteomics analysis of human milk, with the aim of identifying dysregulated proteins in BC vs. control: The milk samples were thawed, and a Bradford assay was conducted to determine total protein concentration in each sample. Then, 800 μg of the proteins for each sample were separated in 11% sodium dodecyl sulfate-polyacrylamide gel electrophoresis (SDS-PAGE) and a Coomassie Blue stained gel was obtained for the milk samples. Each of 12 gel lanes was cut into 30 protein bands, then the bands were excised, cut to very small pieces and underwent in-gel trypsin digestion, as described previously [5]. After overnight in-gel trypsin digestion, the peptides were extracted and purified by Zip-Tip reverse phase chromatography (C18 Ziptip™; Millipore, Billerica, MA, USA). The clean, concentrated peptide mixture was analyzed by nanoLC-MS/MS (a NanoAcquity UPLC coupled with a QTOF Ultima API MS; Waters, Milford, MA, USA), as described elsewhere [16]. The MS raw data from MassLynx software (MassLynx version 4.1, Waters) was converted to peak list (pkl) files by ProteinLynx Global Server software (PLGS version 2.4, Waters) as described elsewhere [17], using the following parameters: a background polynomial of order 5 and a background threshold of 35%, Savitzky-Golay smoothing type, 2 iterations and window of 3 channels, centroid top of 80% of peaks and minimum peak width of 4 channels. The resulting pkl files from PLGS were submitted to our in-house Mascot server (www.matrixscience.com, Matrix Science, London, UK, version 2.5.1) (accessed on 16 October 2022) for protein identification using the following parameters: NCBI_20150706 database (69146588 sequences; 24782014966 residues) (NCBI: national center for biotechnology information), homo sapiens (human) (312165 sequences) as taxonomy, trypsin enzyme, carbamidomethyl (cysteine) as fixed modification, acetylation (lysine), oxidation (methionine), phosphorylation (serine, threonine and tyrosine) as variable modifications, Peptide mass tolerance of ±1.3 Da (one ^13^C isotope), fragment mass tolerance of ±0.8 Da and one maximum missed cleavage. The exported results from Mascot server (in the format of Mascot.DAT files) were then analyzed by the Scaffold software (Scaffold version 4.2.1, Proteome Software Inc., Portland, OR, USA) for statistical analysis of the paired comparison groups and to verify the identified proteins based on the MS/MS data using the following parameters [18]: Protein threshold of minimum 90% probability and minimum two peptides identified by the Protein Prophet algorithm and peptide threshold of minimum 20% probability by the Scaffold Local FDR (false discovery rate) algorithm. To investigate protein dysregulations, the differences with Fisher’s exact test *p*-value ≤ 0.05 and fold change ≥ 2 considered to be statistically significant. Fold change for upregulation (total spectra count of BC divided by total spectra count of control) is shown with positive numbers and fold change for downregulation (spectra count of control sample divided by spectra count of BC sample) is shown with negative numbers.

### 2.4. Data Availability

The data generated during the current study are available from the corresponding author on reasonable request utilizing to Clarkson University’ Material Transfer Agreement.

## 3. Results and Discussion

**Figure 1:**
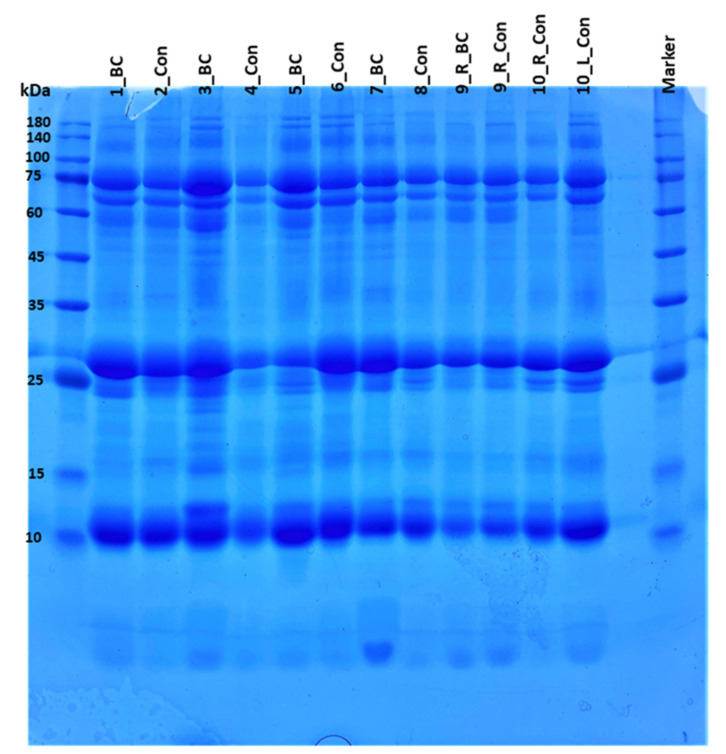
SDS-PAGE of milk samples. One hundred μg of protein was loaded in each well. The molecular weight markers are indicated.

One hundred µg of protein from each of the 12 milk samples comprising the 6 pairs were separated by SDS-PAGE. The gel image is shown in Figure 1. For further proteomics analysis, eight hundred µg of protein from each of the 12 milk samples were separated by SDS-PAGE (Supplementary Materials Figure S1; the lanes in the image were rearranged to present each sample next to its pair). Visual inspection of the 100 μg and 800 μg gel images indicates that the overall protein pattern is very similar among all milk samples. There are however, some differences that can be discerned directly from the gel. For example, both samples from pair 10 (milk from the left and right breasts of a woman who did not have BC, Supplementary Materials Figure S1) lack a major band in the 63 kDa region that is present in both the cancers and controls of the other four pairs. Examination of the results from the database search identifies this region as corresponding to immunoglobulins.

To identify proteins potentially associated with BC, we applied nanoLC-MS/MS analysis on 30 sets of trypsin-digested bands from six pairs of milk samples. As shown in Table 1, the first four pairs included milk from a woman diagnosed with BC and milk from a woman without BC (control or Con). Pairs were constructed to minimize differences in woman’s age, age at first birth, and number of live births. Baby’s age was substantially less for the control samples as compared to the BC samples of the first three pairs. The 5th pair (#9R/L) included milk from the left and right breasts of a woman diagnosed with cancer in only one breast, and the 6th pair included milk from the left and right breasts of a woman with no cancer diagnosis in either breast. This 6th pair (#10L/R) provides a baseline for the number of proteins that can be expected to be differentially expressed in the milk of the left and right breasts of a healthy, non-symptomatic woman.

Analysis using nanoLC-MS/MS revealed several significantly differentially expressed proteins (*p*-value ≤ 0.05 and fold change ≥ 2) among the 5 paired comparisons of BC and control milk samples. Some of the differentially expressed proteins were observed in the single comparison between the milk from left and right breasts of control #10 (woman without cancer). To determine which of the differentially expressed proteins might be markers of BC or BC risk, we identified a subset of these proteins that were similarly dysregulated in our previous studies [5,15] and present them in Table 2, along with information on whether these proteins were differentially expressed in the control comparison (participant 10). Next, we focused only on those proteins for which the differential expression was limited to comparisons between cancer and control (some examples are shown in Supplementary Materials Figure S2a–d).

### 3.1. Differentially Expressed Proteins in BC vs. Control That Were Identified in the Current Study (and Also Identified Erentially Expressed in Our Previous Studies on Human Milk)

Table 2 provides the list of all proteins that were differentially expressed both in our present comparisons of cancer and control breast milk samples. Some of these proteins were also identified in our previous comparisons of cancer and control milk samples [5,15]. Among the proteins differentially expressed between the cancer and control comparisons, some of them were also differentially expressed in the comparison between two control breast milk samples from participant 10 (shaded in Table 2).

Examples of some of the most important dysregulated proteins are shown in Supplementary Materials Figure S2. The spectral count, and fold change of the difference are shown in the graphs. These proteins are important in our comparison study, since the same dysregulation was observed in *multiple comparison pairs* in the current study and observed in our previous studies (mostly on multiple comparison pairs). Additionally, the dysregulation of these proteins did not exist in control samples from right and left breasts of participant 10. These dysregulated proteins include proteins from casein, albumin, lactoferrin and bile salt stimulated lipase families.

Several of the dysregulated proteins were observed in the comparison pair of 3_BC vs. 4_Con (Table 2). In this pair, the BC sample was donated 6.2 years *after* the woman was diagnosed with cancer. The aberrant expression of the proteins related to BC, could either remain or disappear after the cancer is treated, depending on the cause of the dysregulation. This depends on the type of biomarker and whether or not the biomarker has a specific relationship with the therapy [19].

### 3.2. Dysregulated Proteins Specific to the Current Study

In addition to the differentially expressed proteins identified in other studies, we also identified several differentially expressed proteins specific to the current study (Table 2).

For all the protein families in Table 2, here we discuss selected functions, number of milk pairs that showed dysregulation, both in the current study and in our previous studies, and possible role/dysregulation previously found in cancer, based on literature (Table 3). As seen in Table 3, some of these dysregulations were observed in multiple comparison pairs, while others were specific to individual pairs. This is likely because of the wide variety in timing between milk donation and cancer diagnosis across the samples. Additionally, we did the study regardless of subtype of BC in a set of 5 cancer control pairings (small sample group). We still considered these dysregulated proteins, because (based on literature) we found possible relationship between these proteins (or the proteins from the same family or the genes that encode these proteins) and cancer development and in some cases, dysregulation was observed by other research groups, using different methods. The functions of these proteins, as well as the possible relationships between them and cancer are shown in Table 4.

In both the current study and our previous studies [5,15], we observed several protein differences in the *within* woman comparisons of cancer and control (samples 9_R_BC and 9_L_Con in the current study). These differences are important because in this case the differences related to genetic and epigenetics factors between milk samples, which have to be considered in *across* women comparisons, are eliminated. However, when interpreting our paired comparison strategy, it must be considered that the discrepancies in protein dysregulations among different BC vs. control pairs might be due to the wide range in time between milk donation and cancer diagnosis across the samples (as shown in Table 1).

In addition to the dysregulated proteins reported in this study, several immunoglobulins and other components of the immune system were frequently observed to differ between pairs (data not shown). However, we did not observe a consistent pattern between BC and control samples and these data are not discussed here. Varying responses to unrelated responses and to cancer may affect immunoglobulin expression.

## 4. Conclusions

In this study, we performed MS–based proteomics on 12 human milk samples, including 5 paired BC vs. control samples to identify dysregulated proteins in human milk from women with BC vs. control and one comparison group between the right and left breast of a woman without BC to investigate the differences between the protein patterns of milk from different breasts of the same donor. Most of the proteins that we found to be dysregulated in BC vs. control have potential roles in cancer progression and tumor development/ growth and have been shown to be dysregulated in cancer.

Based on our current and published studies [5,15], the tentative draft biomarker signature that we have identified so far contains downregulated Caseins, Bile salt stimulated lipase Xanthine dehydrogenase/oxidase, Lactoferrins, Lactate dehydrogenase, Fatty acid synthase and upregulated Zn–alpha2–glycoprotein and antichymotrypsin. Even if this signature was built from three independent studies, the signature is still fragile because the sample size was small, and our findings must be confirmed in a larger study. Yes, despite all limitations of this and previous studies, our findings support the use of breast milk to examine the BC microenvironment and for BC biomarkers discovery. Therefore, identifying dysregulated proteins in human milk by MS–based proteomics could serve as a tool for detection of BC and assessing BC risk.

## 5. Limitations

This pilot study with 12 milk samples has several limitations. First, we compared the protein profiles of 6 pairs of human milk; a small sample size that could have led to spurious findings. Second, the disparity in baby’s age between the BC and control milk samples could underlie some of the observed differences in protein expression. Third, the time between milk donation and cancer diagnosis varied greatly which effectively made each pair a unique analysis and comparisons across samples difficult. Despite these limitations, some consistencies were observed for proteins differentially expressed in the milk of women with cancer, and these findings support the need for further research.

Another limitation of the current study is the types of proteins that we identified. While we know the identity of most proteins, it is clear to us that more than one protein isoforms are present in the milk samples and identified in the current proteomics study. Yet, it is premature to know which isoproteins are responsible for the onset and/or progression or BC and which isoproteins are actually protecting the breast and preventing BC from developing. Despite this, identifying dysregulated proteins in more than one study and then later identifying additional new proteins demonstrate the power of proteomics in biomarker discovery and warrants further investigation.

## Figures and Tables

**Table 1:** Participants Demographics and Comparison Groups.

Participant	Cancer Diagnosis ER/PR/Her2	Age (Years)	Age at First Birth	Number of Live Births	Baby’s Age (Days)	Family History of BC	Milk Sample Code *	Time of Cancer Diagnosis
1 (2008)	IDC, DCIS Not available	37	34	2	210	yes	1_BC	40 days ***after*** milk donation
2 (2013)	NA	37	34	2	30	yes	2_Con	***NA***
3 (2010)	Carcinoma Not available	43	29	3	570	no	3_BC	6.2 years ***before*** milk donation
4 (2012)	NA	38	32	3	60	no	4_Con	*NA*
5 (2009)	IDC +/+/2+	39	38	1	164	no	5_BC	1 week ***before*** milk donation
6 (2012)	NA	40	40	1	60	yes	6_Con	***NA***
7 (2013)	IDC +/+/−	34	30	2	270	yes	7_BC	5 months ***after*** milk donation
8 (2013)	NA	36	32	2	240	no	8_Con	***NA***
9 (2015)	IDC Not available	38	32	3	600	no	9_R_BC	2 weeks ***before*** milk donation
							9_L_Con	
10 (2015)	NA	33	30	2	180	yes	10_R_Con	***NA***
							10_L_Con	

* Codes for milk. The date after the participant ID indicates the date at which the samples were received at the lab and stored at −20 °C. **IDC** = invasive ductal carcinoma, **DCIS** = ductal carcinoma in situ. **ER/PR/Her2** = estrogen receptor/progesterone receptor/human epidermal growth factor receptor 2. **BC** = milk (combined from left and right breasts) came from a woman diagnosed with **breast cancer. Con** = milk (combined from left and right breasts) came from a woman with no cancer diagnosis; **control. NA** = not applicable. For samples 9 and 10 separate milk samples from the left and right breasts were analyzed; 9_R_BC indicates that the milk came from the right breast of a woman diagnosed with cancer in the right breast; 9_L_Con indicates that the milk came from the left breast (control) of the same woman whose cancer was diagnosed in the right breast, whereas for participant 10 [no BC], each milk sample came from a breast considered a control.

**Table 2:** List of differentially expressed proteins in BC vs. Con.

Protein Family	Identified Protein	Accession Number	Sample Code	Total Spectrum Count		Fold Change	Fisher’s Exact Test (*p*–Value): (*p* ≤ 0.05)
				BC *	Con *		
casein	PREDICTED: alpha–S1–casein isoform X2	gi|578808784 (+1)	1_BC vs. 2_Con	40	8	5	0.00032
			5_BC vs. 6_Con	0	11	–INF	0.034
			7_BC vs. 8_Con	0	8	–INF	0.034
	beta–casein	gi|29674	1_BC vs. 2_Con	0	42	–INF	<0.00010
	Casein alpha s1	gi|118764211	1_BC vs. 2_Con	0	5	–INF	0.011
			5_BC vs. 6_Con	0	11	–INF	0.034
			7_BC vs. 8_Con	0	8	–INF	0.034
	beta–casein isoform 1 precursor	gi|4503087 (+1)	3_BC vs. 4_Con	0	94	–INF	<0.00010
			5_BC vs. 6_Con	6	68	–11.3	<0.00010
			7_BC vs. 8_Con	0	46	–INF	<0.00010
	kappa–casein precursor	gi|148491103 (+2)	5_BC vs. 6_Con	0	37	–INF	<0.00010
			7_BC vs. 8_Con	0	8	–INF	0.034
albumin	alpha–lactalbumin precursor	gi|4504947 (+7)	1_BC vs. 2_Con	7	0	INF	0.026
			5_BC vs. 6_Con	0	11	–INF	0.034
	PRO2675	gi|7770217	3_BC vs. 4_Con	0	52	–INF	<0.00010
			5_BC vs. 6_Con	0	107	–INF	<0.00010
			7_BC vs. 8_Con	0	45	–INF	<0.00010
	albumin	gi|332356380	3_BC vs. 4_Con	0	71	–INF	<0.00010
			5_BC vs. 6_Con	0	228	–INF	< 0.00010
			7_BC vs. 8_Con	16	84	−5.2	< 0.00010
	serum albumin	gi|62113341	3_BC vs. 4_Con	0	68	–INF	< 0.00010
			5_BC vs. 6_Con	0	217	–INF	<0.00010
			7_BC vs. 8_Con	0	82	–INF	<0.00010
	serum albumin	gi|28592	5_BC vs. 6_Con	0	217	–INF	<0.00010
			9_R_BC vs. 9_L_Con	0	111	–INF	<0.00010
	Chain A, Human Serum Albumin Complexed with Myristate and Aspirin	gi|122920512	5_BC vs. 6_Con	0	229	–INF	<0.00010
	serum vitamin D–binding protein precursor (a member of albumin family)	gi|181482 (+2)	9_R_BC vs. 9_L_Con	5	0	INF	0.036
antichymotrypsin	alpha–1–antichymotrypsin	gi|177809 (+1)	7_BC vs. 8_Con	8	3	2.7	0.01
	Chain A, Crystal Structure of Cleaved Human Alpha1–antichymotrypsin at 2.7 Angstroms Resolution and Its Comparison with Other Serpins	gi|443345	7_BC vs. 8_Con	9	0	INF	<0.00010
			10_R_Con vs. 10_L_Con	10	0	INF	0.00023
Zn–alpha2–glycoprotein	Zn–alpha2–glycoprotein	gi|38026	3_BC vs. 4_Con	5	0	INF	0.00026
			10_R_Con vs. 10_L_Con	6	0	INF	0.0066
lactoferrin	lactoferrin	gi|193527456	3_BC vs. 4_Con	0	459	–INF	<0.00010
			5_BC vs. 6_Con	0	592	–INF	<0.00010
			7_BC vs. 8_Con	0	217	–INF	<0.00010
	lactoferrin	gi|58372399	3_BC vs. 4_Con	0	442	–INF	<0.00010
			5_BC vs. 6_Con	109	583	−5.3	<0.00010
	Chain A, R210k N–Terminal Lobe Human Lactoferrin	gi|7245541	3_BC vs. 4_Con	0	261	–INF	<0.00010
			5_BC vs. 6_Con	0	335	–INF	<0.00010
	Chain A, Structure of Human Diferric Lactoferrin At 2.5a Resolution Using Crystals Grown at Ph 6.5	gi|48425709	3_BC vs. 4_Con	0	382	–INF	<0.00010
			5_BC vs. 6_Con	0	494	–INF	<0.00010
			7_BC vs. 8_Con	0	173	–INF	<0.00010
	Lactotransferrin	gi|18490850	3_BC vs. 4_Con	0	455	–INF	<0.00010
			5_BC vs. 6_Con	0	590	–INF	<0.00010
	Chain A, Molecular Replacement Solution of The Structure of Apolactoferrin, A Protein Displaying Large–Scale Conformational Change	gi|157831799	5_BC vs. 6_Con	113	575	−5.1	<0.00010
	lactoferrin precursor	gi|12083188 (+1)	5_BC vs. 6_Con	111	583	−5.2	<0.00010
			7_BC vs. 8_Con	0	217	–INF	<0.00010
	lactoferrin	gi|38154680	5_BC vs. 6_Con	103	553	−5.4	<0.00010
			7_BC vs. 8_Con	0	209	–INF	<0.00010
bile salt stimulated lipase	carboxyl ester lipase (bile salt–stimulated lipase), isoform CRA_b, partial	gi|119608437	3_BC vs. 4_Con	0	156	–INF	<0.00010
			5_BC vs. 6_Con	22	191	−8.7	<0.00010
			7_BC vs. 8_Con	0	105	–INF	<0.00010
	carboxyl ester lipase (bile salt–stimulated lipase), isoform CRA_c	gi|119608438	3_BC vs. 4_Con	0	111	–INF	<0.00010
	Chain A, Structure of The Catalytic Domain of Human Bile Salt Activated Lipase	gi|11514505	3_BC vs. 4_Con	23	160	−7	0.0085
			5_BC vs. 6_Con	21	192	−9.1	<0.00010
			7_BC vs. 8_Con	26	105	−4	<0.00010
xanthine dehydrogenase	xanthine dehydrogenase	gi|984267	3_BC vs. 4_Con	0	78	–INF	<0.00010
			5_BC vs. 6_Con	0	93	–INF	<0.00010
			7_BC vs. 8_Con	0	22	–INF	<0.00010
			10_R_Con vs. 10_L_Con	121	44	2.75	<0.00010
	Chain A, Crystal Structure of Human Xanthine Oxidoreductase Mutant, Glu803val	gi|158428225 (+1)	5_BC vs. 6_Con	19	97	−5.1	0.0062
			10_R_Con vs. 10_L_Con	124	45	2.76	<0.00010
fatty acid synthase	FASN variant protein	gi|68533031	3_BC vs. 4_Con	0	41	–INF	0.00014
			5_BC vs. 6_Con	18	84	−4.7	0.023
			7_BC vs. 8_Con	37	18	2.1	<0.00010
			10_R_Con vs. 10_L_Con	65	16	4.1	<0.00010
	fatty acid synthase	gi|41584442	3_BC vs. 4_Con	0	40	–INF	0.00018
			5_BC vs. 6_Con	0	80	–INF	<0.00010
			10_R_Con vs. 10_L_Con	65	0	INF	<0.00010
	encodes region of fatty acid synthase activity; FAS; multifunctional protein	gi|1049053	5_BC vs. 6_Con	0	63	–INF	<0.00010
			10_R_Con vs. 10_L_Con	46	13	3.5	<0.00010
	Chain A, Enoyl–acyl Carrier Protein–reductase Domain from Human Fatty Acid Synthase	gi|697351654	5_BC vs. 6_Con	0	13	–INF	0.018
			7_BC vs. 8_Con	9	0	INF	<0.00010
			10_R_Con vs. 10_L_Con	12	0	INF	<0.00010
	Chain A, Crystal Structure of The Human Fatty Acid Synthase Thioesterase Domain with an Activate Site–Specific Polyunsaturated Fatty Acyl Adduct	gi|347948699	9_R_BC vs. 9_L_Con	0	33	–INF	<0.00010
mannose receptor	mannose receptor	gi|109895388	3_BC vs. 4_Con	0	28	–INF	0.0024
			5_BC vs. 6_Con	0	40	–INF	<0.00010
			7_BC vs. 8_Con	0	31	–INF	<0.00010
			9_R_BC vs. 9_L_Con	51	20	2.5	0.00032
			10_R_Con vs. 10_L_Con	69	32	2.1	<0.00010
fatty acid–binding protein	fatty acid–binding protein, heart isoform 2	gi|4758328 (+6)	3_BC vs. 4_Con	0	21	–INF	0.011
			5_BC vs. 6_Con	0	12	–INF	0.025
zinc finger protein	zinc finger protein 292	gi|150170718	5_BC vs. 6_Con	3	0	INF	0.018
	bassoon (Zinc finger protein 231) (presynaptic cytomatrix protein), isoform CRA_a	gi|119585396 (+1)	9_R_BC vs. 9_L_Con	0	7	–INF	0.0065
	CXXC–type zinc finger protein 5 [Homo sapiens]	gi|158261990	10_R_Con vs. 10_L_Con	7	0	INF	0.019
adipophilin	adipophilin	gi|1806040 (+2)	5_BC vs. 6_Con	0	30	–INF	<0.00010
			7_BC vs. 8_Con	5	0	INF	0.0046
			10_R_Con vs. 10_L_Con	43	10	4.3	<0.00010
apolipoprotein	apolipoprotein J precursor	gi|178855 (+4)	5_BC vs. 6_Con	0	10	–INF	0.046
			10_R_Con vs. 10_L_Con	23	7	3.3	0.00021
actin	gamma–actin, partial	gi|178045	9_R_BC vs. 9_L_Con	0	13	–INF	<0.00010
titin	titin isoform IC	gi|642945631	9_R_BC vs. 9_L_Con	0	11	–INF	0.00036
S100 family	Hornerin	gi|57546919	9_R_BC vs. 9_L_Con	0	5	–INF	0.027
			10_R_Con vs. 10_L_Con	6	0	INF	0.033
stomatin	band 7.2b stomatin	gi|1103842	9_R_BC vs. 9_L_Con	5	0	INF	0.036
lactadherin	PREDICTED: lactadherin isoform X1	gi|530407155	1_BC vs. 2_Con	8	0	INF	0.016
			3_BC vs. 4_Con	0	23	–INF	0.0071
			5_BC vs. 6_Con	0	28	–INF	0.00018
	lactadherin isoform a preproprotein	gi|167830475	1_BC vs. 2_Con	8	0	INF	0.016
			3_BC vs. 4_Con	0	35	–INF	0.00053
			5_BC vs. 6_Con	0	33	–INF	<0.00010
O–linked N–acetylglucosamine (GlcNAc) transferase	Chain E, Human O–Glcnac Transferase (Ogt) In Complex with Udp–5sglcnac Additionally, Substrate Peptide	gi|409973764	3_BC vs. 4_Con	3	0	INF	0.0071
enolase	gamma–enolase	gi|5803011 (+6)	3_BC vs. 4_Con	2	0	INF	0.037
galactosyltransferase	beta–1,4–galactosyltransferase 1	gi|13929462	5_BC vs. 6_Con	4	0	INF	0.0048
recoverin	Chain A, Crystal Structure of Human Recoverin At 2.2 A Resolution	gi|134104098	5_BC vs. 6_Con	3	0	INF	0.018
NADH dehydrogenase	NADH dehydrogenase subunit 5 (mitochondrion)	gi|416949295	5_BC vs. 6_Con	3	0	INF	0.018
	NADH dehydrogenase subunit 5, partial (mitochondrion)	gi|416949335	9_R_BC vs. 9_L_Con	6	0	INF	0.018
	NADH dehydrogenase subunit 5 (mitochondrion)	gi|381243849	10_R_Con vs. 10_L_Con	6	0	INF	0.033
perilipin	perilipin–3 isoform 1	gi|255958282 (+1)	5_BC vs. 6_Con	3	0	INF	0.018
			7_BC vs. 8_Con	4	0	INF	0.014
tRNA synthetase–tRNA complex	Chain A, Charged and Uncharged Trnas Adopt Distinct Conformations When Complexed with Human Tryptophanyl–Trna Synthetase	gi|112490030	5_BC vs. 6_Con	3	0	INF	0.018
histone–lysine methyltransferase	histone–lysine N–methyltransferase SETD2	gi|197313748 (+3)	5_BC vs. 6_Con	3	0	INF	0.018
UTP––glucose–1–phosphate uridylyltransferase	UTP––glucose–1–phosphate uridylyltransferase isoform a	gi|48255966 (+3)	5_BC vs. 6_Con	0	16	–INF	0.0072
ribosomal protein	40S ribosomal protein S5	gi|13904870 (+3)	7_BC vs. 8_Con	3	0	INF	0.04
human protein disulfide isomerase (Hpdi)	Chain A, Crystal Structure of Reduced Hpdi (abb’xa’)	gi|478247271	9_R_BC vs. 9_L_Con	11	0	INF	0.00064
			10_R_Con vs. 10_L_Con	5	0	INF	0.015
elongation factor	elongation factor 2	gi|4503483	9_R_BC vs. 9_L_Con	7	0	INF	0.0093
clathrin	clathrin heavy chain1 isoform1	gi|4758012 (+8)	9_R_BC vs. 9_L_Con	9	2	4.5	0.039

* The BC and Con designations apply to milk samples from women 1–9; samples from woman 10 are both controls (no cancer). Gray background: They are within woman comparison.

**Table 3:** Protein functions, type of dysregulation, number of pairs that showed dysregulation and possible role/dysregulation, previously found in cancer based on literature for the proteins discussed in Table 2.

Protein Family	Dysregulation in the Current Study	Dysregulation in Our Previous Studies on Human Milk	Selected Functions	Cancer Related Investigations
casein	- Eleven downregulations in 4 out of 5 pairs - One upregulation, in 1_BC vs. 2_Con	- Seven downregulations in 4 out of 5 pairs [5]	- Transportation of calcium phosphate - Playing a role in growth by providing essential amino acids - Antioxidant activity	- Downregulated in human tumor tissues including BC tumors [20,21]. - Downregulated in prostate cancer (and normal prostate tissue) vs. benign prostate hyperplasia [22].
albumin	- Thirteen downregulations in 4 out of 5 pairs - Two upregulations, in 1_BC vs. 2_Con and 9_R_BC vs. 9_L_Con	- Three downregulations in one pair of *within* woman comparison [15]	- Main protein in blood which maintains osmotic pressure by binding to other molecules and performing transportation in blood	- Downregulation is reported in serum of patients with carcinomas of unknown primary sites [23,24]
lactoferrin	- Seventeen downregulations in 3 out of 5 pairs	- Eleven downregulations in one pair of *within* woman comparison [15]	- Involved in transcription	- Low levels were reported in BC [25,26]. - Low levels of lactoferrin mRNA observed in both cancer cell lines and tumors [27]. - Downregulation of both mRNA and protein reported in BC patients [25]. - The levels of the protein could be different based on the subtype of BC. (lower levels observed in ER–negative compared to ER–positive) [28].
bile salt–stimulated lipase	- Seven downregulations in 3 out of 5 pairs	- Two downregulations in 2 out of 5 pairs [5]	- Involved in fat digestion	- Low expression of the gene has been observed in the bile acids synthesis pathway in BC tumor tissues [29].
xanthine dehydrogenase	- Four downregulations in 3 out of 5 pairs - Two dysregulations in control samples from participant 10	- Three downregulations in 3 out of 5 pairs [5]	- Involved in purine catabolism	- Downregulation observed in BC patients [30]. - Involved in uric acid synthesis (which has antioxidant activity) [31].
mannose receptor	- Three downregulations in 3 out of 5 pairs - One upregulation in 1 out of 5 pairs - One dysregulation in control samples from participant 10	- Two downregulations in 2 out of 5 pairs [5]	- Involved in microphage migration	- Could be involved in tumor progression because of its role in microphage migration [32,33].
antichymotrypsin	- Two upregulations in 1 out of 5 pairs - One dysregulation in control samples from participant 10	- Five upregulations in 3 out of 5 pairs [5]	- A protease inhibitor that protects tissues from enzymatic damage	- The gene might be involved in cancer development [34]. - Upregulated in lung cancer tissues [35]. - Upregulated in prostate cancer tissues [36].
Zn–alpha2–glycoprotein	- One upregulation in 1 out of 5 pairs - One dysregulation in control samples from participant 10	- One upregulation in one pair of *within* woman comparison [15]	- Lipid degradation - In high levels, could cause body fat deficiency and cachexia	- Reported to be a potential biomarker in different cancers, including BC [37]. - Upregulated in BC tumors [38]. - Upregulated in advanced BC tumors [39]. - High gene expression has been reported in BC [40].
fatty acid synthase	- Seven downregulations in 3 out of 5 pairs - Four dysregulations in control samples from participant 10	- Five downregulations in 5 out of 5 pairs [5]	- Enzyme for fatty acids synthesis	- Upregulated in different cancers including BC [41]. - Upregulated in serum samples of patients with BC [42,43] - Upregulated in serum samples of patients with BC as well as BC cell lines [44].
fatty acid–binding protein	- Two downregulations in 2 out of 5 pairs	- One downregulation in one pair of *within* woman comparison [15]	- Involved in metabolism of fatty acids	- Downregulated in BC cell lines [45,46]. - Downregulated in prostate cancer tumors and cell lines [47].
zinc finger protein	- One upregulation in 1 out of 5 pairs - One downregulation in 1 out of 5 pairs - One dysregulation in control samples from participant 10	- One upregulation in 1 out of 5 pairs [5]	- Involved in transcription	- Upregulation of the gene and protein of bromodomain PHD finger transcription factor (from the same family) has been reported in colorectal cancer [48,49].
adipophilin	- One upregulation in 1 out of 5 pairs - One downregulation in 1 out of 5 pairs - One dysregulation in control samples from participant 10	- One upregulation in 1 out of 5 pairs [5]	- Involved in adipose differentiation	- Upregulated in different cancers [50]. - Upregulated in tumor tissues of hepatocellular cancer [51,52].
apolipoprotein	- One downregulation in 1 out of 5 pairs - One dysregulation in control samples from participant 10	- Eight downregulations in one pair of *within* woman comparison [15]	- Involved in lipid transportation	- Downregulated in human adenoid cystic carcinoma [53].
actin	- One downregulation in 1 out of 5 pairs	- One upregulation in 1 out of 5 pairs [5]	- Involved in cellular processes	- Involved in tumor development [54,55].
titin	- One downregulation in 1 out of 5 pairs	- Three downregulation in 3 out of 5 pairs and one upregulation in 1 out of 5 pairs [5]	- Involved in muscle function	- Gene alteration has been reported to be related to BC risk [56,57]
S100 family	- One downregulation in 1 out of 5 pairs - One dysregulation in control samples from participant 10	- Two downregulations in one pair of *within* woman comparison [15]	- Involved in cellular processes	- Involved in cancer development and have shown dysregulations in different cancers [58] - Low levels have been reported to be related to cancer development [59]
Stomatin	- One upregulation in 1 out of 5 pairs	- One upregulation in 1 out of 5 pairs [5]	- Cell membrane protein, might be involved in ion channels transportations.	- Upregulation is reported in ovarian cancer [60,61]

**Table 4:** Protein functions, type of dysregulation, number of milk pairs that showed dysregulation and possible role/dysregulation, previously found in cancer based on literature for the proteins discussed in Table 3.

Protein Family	Dysregulation in the Current Study	Selected Functions	Cancer Related Investigations
lactadherin	Four downregulations in 2 out of 5 pairs Two upregulations in 1 out of 5 pairs	- Involved in cell adhesion and neovascularization	Downregulated in ER positive BC progression, although upregulated in triple negative BC [62]. High expression of MFG–E8 (gene that encodes lactadherin) observed in breast carcinomas [63].
O–linkedN–acetyl Glucosamine transferase (GlcNAc)	One upregulation in 1 out of 5 pairs	- Enzyme involved in protein glycosylation	Upregulated in cancers (including BC) and is involved in cancer progression [64]. Upregulated in BC and plays a role in cancer cells glycolysis [65]. Upregulated in BC cell lines [66]. Upregulated in prostate cancer cell lines [67]. Upregulated in lung and colon cancer tissues [68].
Enolase	One upregulation in 1 out of 5 pairs	- Enzyme involved in glycolysis	Upregulated in different types of cancers including BC [69,70]. Elevated levels in BC, resulted from environmental pollutants [71]. Upregulated in BC tissues [72].
galactosyltransferase	One upregulation in 1 out of 5 pairs	- Enzyme for galactose transfer	Plays a role in BC cell line proliferation [73]. Plays a role in cell adhesion in BC cell line [74]. Plays a role in cell transformation to malignancy [75]. Upregulated in malignant BC tissues and cell lines [75]. Upregulated in lung cancer cells [75,76,77].
recoverin	One upregulation in 1 out of 5 pairs	- Ca2+ sensor, involved in visual response	Altered levels have been reported in different cancers including BC [78]. Based on NCBI, Plays a role in retia damage, caused by cancer [79].
NADH dehydrogenase	Two upregulations in 2 out of 5 pairs One dysregulation in control samples from participant 10	- Enzyme involved in ATP synthesis	Gene polymorphisms happen in BC patients [80,81,82].
perilipin	Two upregulations in 2 out of 5 pairs	- Involved in lipid metabolism	Plays a role in cancer development [83]. Highly expressed in BC based on the Human Protein Atlas [84].
tRNA synthetase–tRNA complex	One upregulation in 1 out of 5 pairs	- Involved in protein synthesis	Tryptophanyl–tRNA synthetase has been reported to be upregulated in BC tumors [85]. Tryptophanyl–tRNA synthetase is highly expressed in BC based on the Human Protein Atlas [86]
histone–lysine methyltransferase	One upregulation in 1 out of 5 pairs	- Catalyzes methyl transfer to lysine residue in histones which is important in gene expression and cell division	Plays a role in BC development and is dysregulated in BC [87].
UTP––glucose–1–phosphate uridylyltransferase	One downregulation in 1 out of 5 pairs	- Involved in metabolism of carbohydrates	Downregulated in different types of tumors [88,89]. Lower expression in BC based on the Human Protein Atlas [90].
ribosomal protein	One upregulation in 1 out of 5 pairs	- Involved in protein translation	Play a role in tumor development and has shown altered levels in different cancers [91]. Upregulated in mice mammary gland tumors [92]. Upregulated in M4A4 BC cell line [93]
human protein disulfide isomerase (Hpdi)	One upregulation in 1 out of 5 pairs One dysregulation in control samples from participant 10	- Enzyme involved in protein folding	Involved in cancer development and progression [94]. Upregulated in different types of cancers [95].
elongation factor	One upregulation in 1 out of 5 pairs	- Plays a role in cell cycle and protein translation	Upregulation has been reported in different cancers [96,97] Overexpression is reported in BC tumors [98]
clathrin	One upregulation in 1 out of 5 pairs	- Involved in coated vesicles formation	High expression has been reported in BC based on the Human Protein Atlas [99,100].

## Data Availability

Any data from this manuscript can be requested and is available upon request to CCD.

## Supplementary Materials

The following supporting information can be downloaded at: https://www.mdpi.com/article/10.3390/proteomes10040036/s1, Figure S1: SDS-PAGE of milk samples. Eight hundred μg of protein was loaded in each well. For better understanding, the gel lanes were cropped, and comparison pairs are shown next to each other; Figure S2. Dysregulated proteins in BC vs. control, also found to be dysregulated in our previous studies on human milk, which did not show any dysregulations in control samples from participant 10. Each bar graph shows total spectra counting in BC (in red) vs. control (in blue) for different proteins *within* the same family. The bars are labeled by the corresponding comparison pair and the fold change (FC) for each comparison. The red label means that the corresponding pair showed inconsistency compared to the other pairs in terms of up or down regulation.

## Abbreviations

BC, breast cancer; SDS–PAGE, sodium dodecyl sulfate–polyacrylamide gel electrophoresis; 2D–PAGE, two–dimensional polyacrylamide gel electrophoresis; 1D, one–dimensional; MS, mass spectrometry; MS/MS, tandem mass spectrometry; nanoLC–MS/MS, nanoliquid chromatography tandem mass spectrometry; pkl, peak list; PLGS, ProteinLynx Global Server; NCBI, National Center for Biotechnology Information; Gi, GenInfo identifier; FDR, false discovery rate.
