# Supplementary material for: Mass Spectrometry-Based Proteomics of Human Milk to Identify Differentially Expressed Proteins in Women with Breast Cancer versus Controls
Source: Proteomes. 2022 Oct 28;10(4):36. doi: 10.3390/proteomes10040036 (PMC9680319; doi:10.3390/proteomes10040036)
Supplement: Supplementary file 1 [file proteomes-10-00036-s001.zip › proteomes-1921028-supplementary.pdf]

proteomes

MDPI

Article

Mass Spectrometry-Based Proteomics of Human Milk to Identify Differentially Expressed Proteins in Women with Breast Cancer versus Controls

Roshanak Aslebagh [1], Danielle Whitham [1], Devika Channaveerappa [1], Panashe Mutsengi [1], Brian Pentecost [2], Kathleen F. Arcaro [2] and Costel C. Darie [1,3]

[1] Biochemistry & Proteomics Group, Department of Chemistry & Biomolecular Science, Clarkson University, 8 Clarkson Avenue, Potsdam, NY, 13699-5810, USA

[2] Department of Veterinary & Animal Sciences, University of Massachusetts, Amherst, 240 Thatcher Road, LSL 1 Room N529, Amherst, MA 01003-9298

* Correspondence: Costel C. Darie, Tel: (315) 268-7763, Fax: (315) 268-6610, E-mail: cdarie@clarkson.edu

Citation: Aslebagh, R.; Whitham, D.; Channaveerappa, D.; Mutsengi, P.; Pentecost, B.; Arcaro, K.F.; Darie, C.C. Mass Spectrometry-Based Proteomics of Human Milk to Identify Differentially Expressed Proteins in Women with Breast Cancer versus Controls. *Proteomes* **2022**, *10*, 36. https://doi.org/10.3390/proteomes10040036

Academic Editors: Rodrigo Barderas-Manchado and Thomas Kislinger

Received: 1 September 2022

Accepted: 25 October 2022

Published: 28 October 2022

Publisher's Note: MDPI stays neutral with regard to jurisdictional claims in published maps and institutional affiliations.

Copyright: © 2022 by the authors. Licensee MDPI, Basel, Switzerland. This article is an open access article distributed under the terms and conditions of the Creative Commons Attribution (CC BY) license (https://creativecommons.org/licenses/by/4.0/).

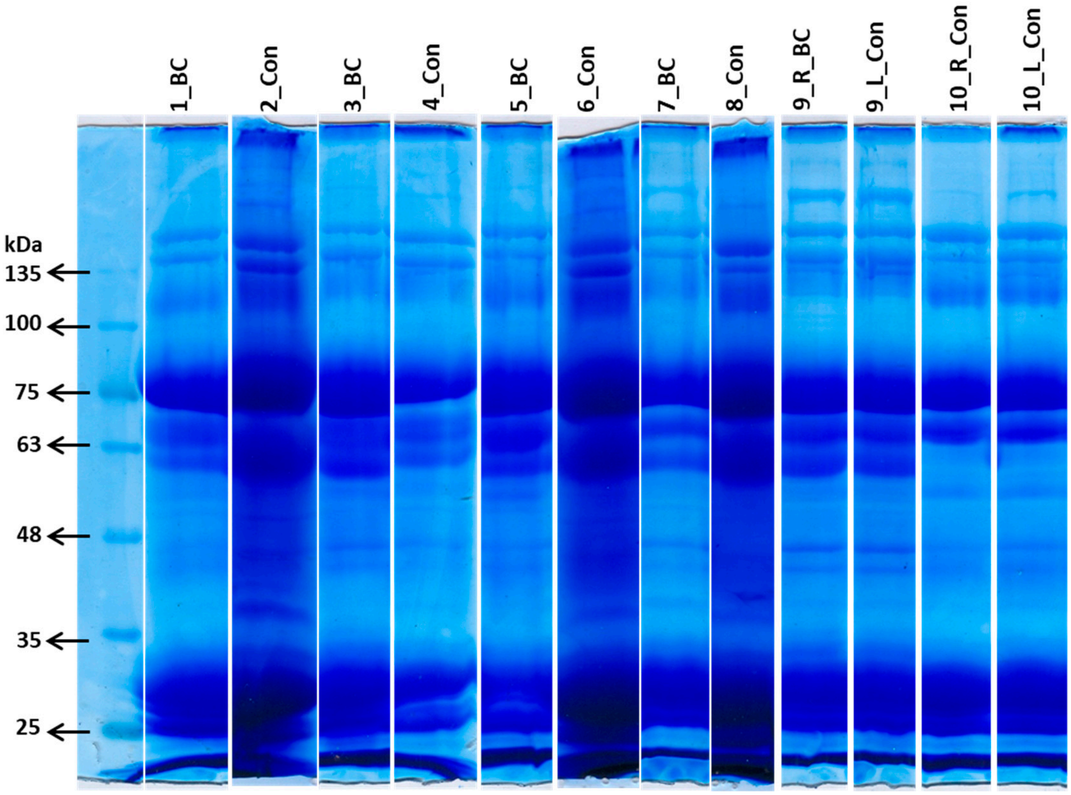

Supplemental Figure S1. SDS-PAGE of milk samples. Eight hundred μg of protein was loaded in each well. For better understanding, the gel lanes were cropped, and comparison pairs are shown next to each other.

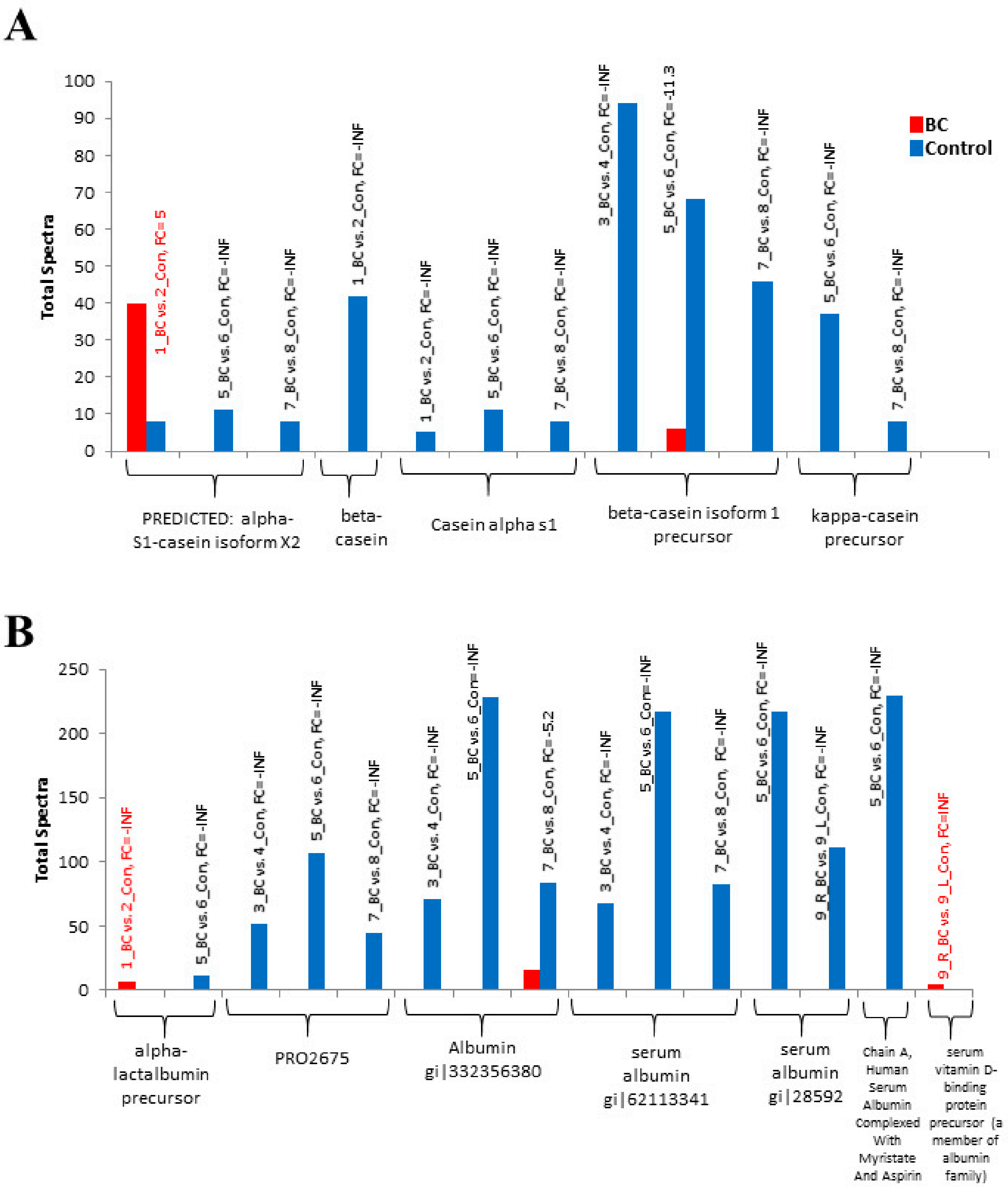
A
Total Spectra
BC
Control
1_BC vs. 2_Con, FC= 5
5_BC vs. 6_Con, FC=-INF
7_BC vs. 8_Con, FC=-INF
1_BC vs. 2_Con, FC=-INF
1_BC vs. 2_Con, FC=-INF
5_BC vs. 6_Con, FC=-INF
7_BC vs. 8_Con, FC=-INF
3_BC vs. 4_Con, FC=-INF
5_BC vs. 6_Con, FC=-11.3
7_BC vs. 8_Con, FC=-INF
5_BC vs. 6_Con, FC=-INF
7_BC vs. 8_Con, FC=-INF
PREDICTED: alpha-S1-casein isoform X2
beta-casein
Casein alpha s1
beta-casein isoform 1 precursor
kappa-casein precursor
B
Total Spectra
1_BC vs. 2_Con, FC=-INF
5_BC vs. 6_Con, FC=-INF
3_BC vs. 4_Con, FC=-INF
5_BC vs. 6_Con, FC=-INF
7_BC vs. 8_Con, FC=-INF
3_BC vs. 4_Con, FC=-INF
5_BC vs. 6_Con=-INF
7_BC vs. 8_Con, FC=-5.2
3_BC vs. 4_Con, FC=-INF
5_BC vs. 6_Con=-INF
7_BC vs. 8_Con, FC=-INF
5_BC vs. 6_Con, FC=-INF
9_R_BC vs. 9_L_Con, FC=-INF
5_BC vs. 6_Con, FC=-INF
9_R_BC vs. 9_L_Con, FC=INF
alpha-lactalbumin precursor
PRO2675
Albumin gi|332356380
serum albumin gi|62113341
serum albumin gi|28592
Chain A, Human Serum Albumin Complexed With Myristate And Aspirin
serum vitamin D-binding protein precursor (a member of albumin family)

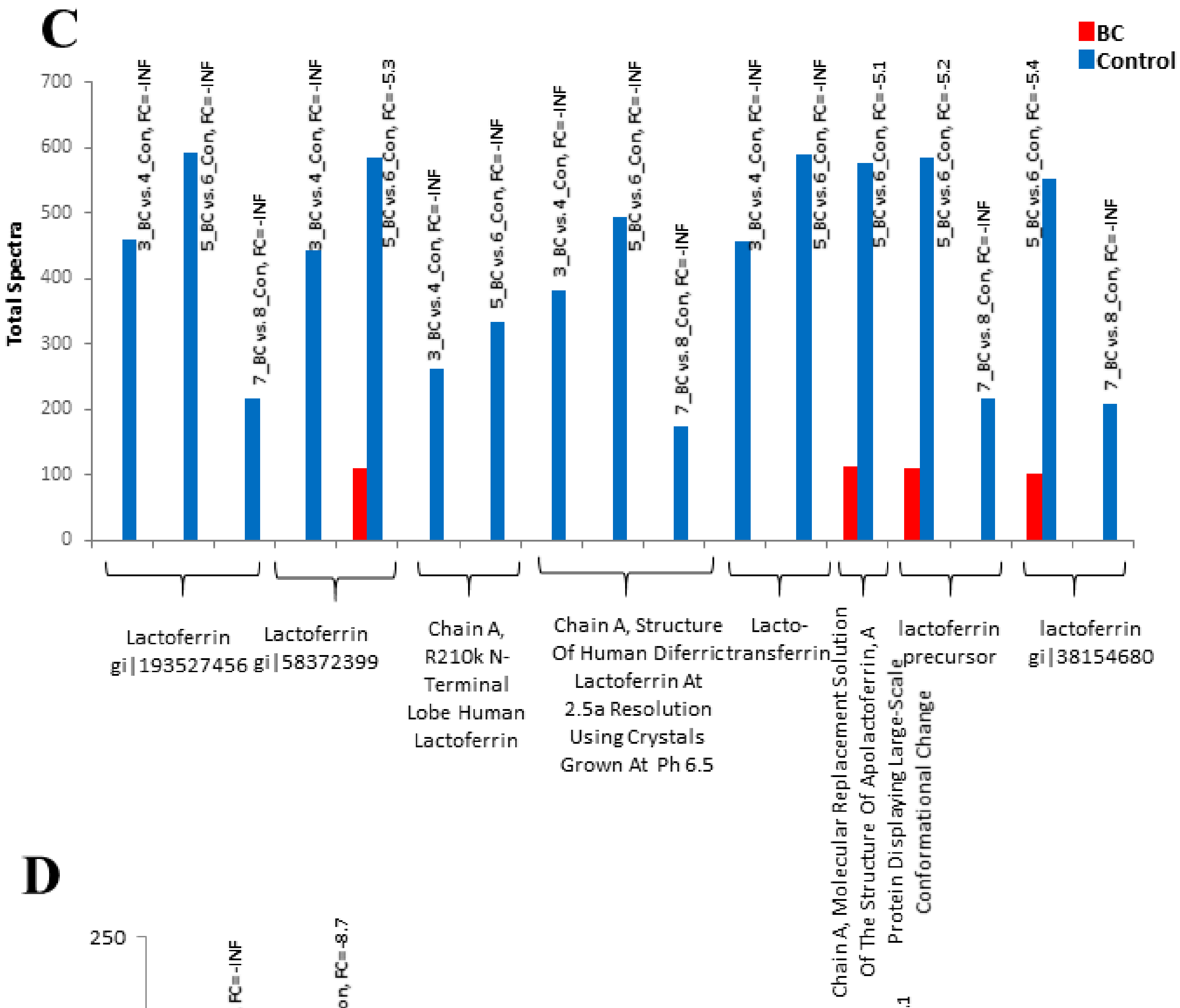
C
BC
Control
Total Spectra
700
600
500
400
300
200
100
0
3_BC vs. 4_Con, FC=-INF
5_BC vs. 6_Con, FC=-INF
7_BC vs. 8_Con, FC=-INF
3_BC vs. 4_Con, FC=-INF
5_BC vs. 6_Con, FC=-5.3
3_BC vs. 4_Con, FC=-INF
5_BC vs. 6_Con, FC=-INF
3_BC vs. 4_Con, FC=-INF
5_BC vs. 6_Con, FC=-INF
7_BC vs. 8_Con, FC=-INF
3_BC vs. 4_Con, FC=-INF
5_BC vs. 6_Con, FC=-INF
5_BC vs. 6_Con, FC=-5.1
5_BC vs. 6_Con, FC=-5.2
7_BC vs. 8_Con, FC=-INF
5_BC vs. 6_Con, FC=-5.4
7_BC vs. 8_Con, FC=-INF
Lactoferrin gi|193527456
Lactoferrin gi|58372399
Chain A, R210k N-Terminal Lobe Human Lactoferrin
Chain A, Structure Of Human Diferric Lactoferrin At 2.5a Resolution Using Crystals Grown At Ph 6.5
Lacto-transferrin
Chain A, Molecular Replacement Solution Of The Structure Of Apolactoferrin, A Protein Displaying Large-Scale Conformational Change
lactoferrin precursor
lactoferrin gi|38154680

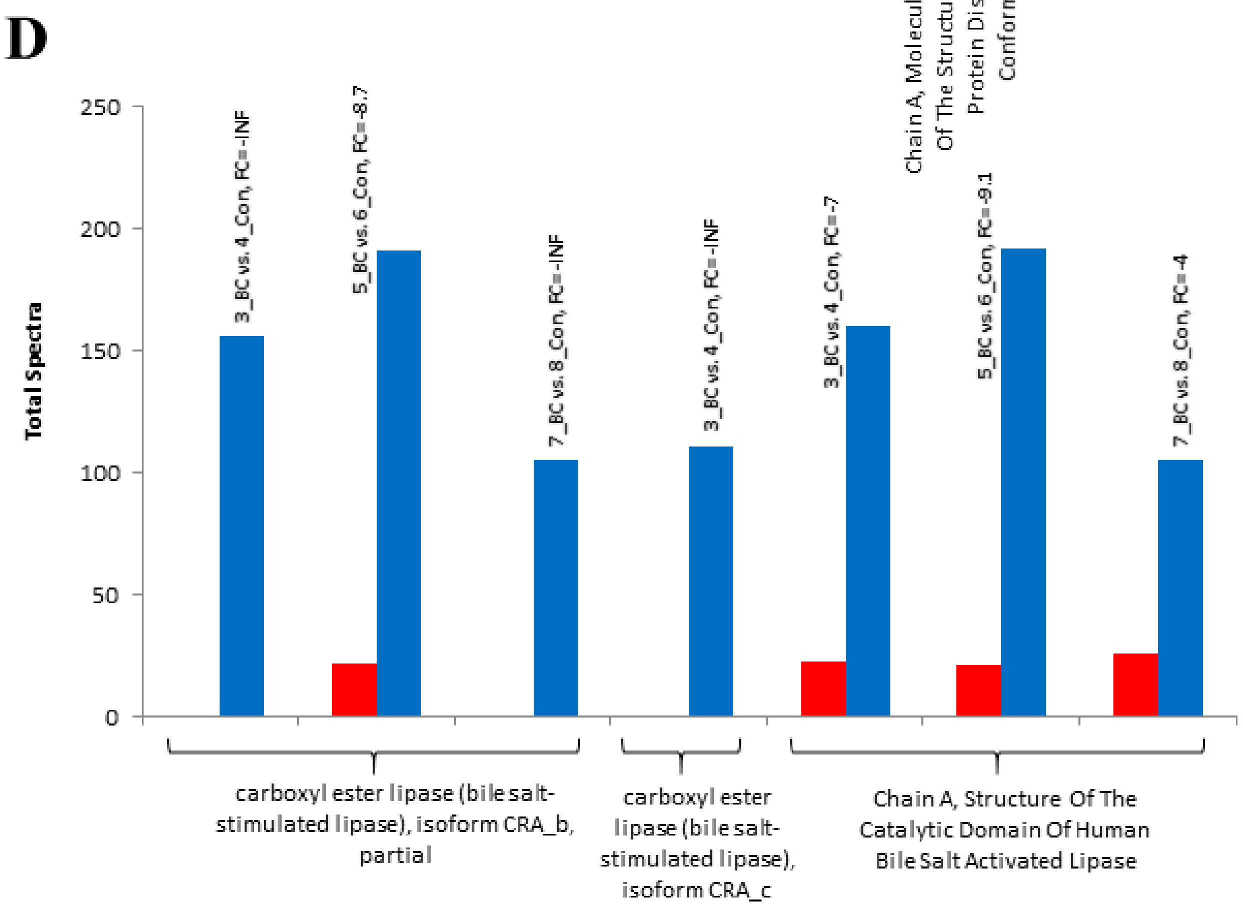
D
Total Spectra
250
200
150
100
50
0
3_BC vs. 4_Con, FC=-INF
5_BC vs. 6_Con, FC=-8.7
7_BC vs. 8_Con, FC=-INF
3_BC vs. 4_Con, FC=-INF
3_BC vs. 4_Con, FC=-7
5_BC vs. 6_Con, FC=-9.1
7_BC vs. 8_Con, FC=-4
carboxyl ester lipase (bile salt-stimulated lipase), isoform CRA_b, partial
carboxyl ester lipase (bile salt-stimulated lipase), isoform CRA_c
Chain A, Structure Of The Catalytic Domain Of Human Bile Salt Activated Lipase

Supplemental Figure S2. Dysregulated proteins in BC vs. control, also found to be dysregulated in our previous studies on human milk, which did not show any dysregulations in control samples from participant 10. Each bar graph shows total spectra counting in BC (in red) vs. control (in blue) for different proteins ***within*** the same family. The bars are labeled by the corresponding comparison pair and the fold change (FC) for each comparison. The red label means that the corresponding pair showed inconsistency compared to the other pairs in terms of up or down regulation. **a:** Downregulation of the proteins from casein family in BC vs. control. **b:** Downregulation of the proteins from albumin family in BC vs. control. **c:** Downregulation of the proteins from lactoferrin family in BC vs. control. **d:** Downregulation of the proteins from bile salt stimulated lipase family in BC vs. control.
